# Primary total knee arthroplasty in severe valgus knee

**Published:** 2013-12-25

**Authors:** R Radulescu, A Badila, I Japie, T Ciobanu, R Manolescu

**Affiliations:** * ”Carol Davila” University of Medicine and Pharmacy, Bucharest; ** Department of Orthopedic Surgery, Bucharest University Emergency Hospital, Romania

**Keywords:** valgus knee, arthroplasty, osteoarthritis

## Abstract

Abstract

Aim: Outcome of primary total arthroplasty for osteoarthritis of the knee with valgus deformity.

Materials and methods: Between 2005 and 2007, 28 primary total knee replacements were performed for osteoarthritis of the knee with valgus deformity. 21 cases were women and 7 men with a mean age of 66.6 years (extremes 54-81). The clinical and radiological evaluations were done considering the knee range of motion, Knee Society Score (KSS) and femorotibial angle measured on the frontal standing long leg X-rays. Preoperatively, the knee valgus deformity angle was 6 to 15 degrees in 14 cases, 15 to 25 degrees in 10 cases and over 25 degrees in 4 cases.

Results: After a mean follow-up time of 14 months (extremes 7-29), the knee range of motion improved from a mean of 71 degrees (extremes 52-87) preoperatively to a mean of 95 degrees (extremes 78-110) postoperatively. The KSS value improved from 21.3 points (extremes 1-33) preoperatively to 80.7 points (extremes 70-92) postoperatively and the frontal femorotibial angle from a mean value of 21 degrees (extremes 11-39) of valgus before surgery, to a mean of 9 degrees (extremes 0-12) of valgus after surgery.

Conclusions: Long leg AP view X-ray examination in standing position is mandatory. The standard medial parapatellar approach is appropriate in this type of arthroplasty even if significant knee valgus deviations are present because it avoids the lateral approach complications. Postoperatively, one can get an aligned and stable knee if a judicious and progressive periarticular soft tissues balancing is achieved, in both flexion and extension position.

## Introduction

**Fig. 1:**
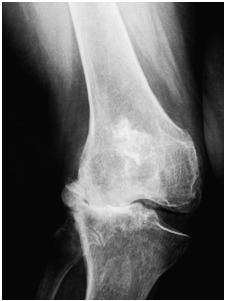
The anatomical changes of the lateral compartment in valgus knee

From the total knee arthroplasty point of view, the valgus deformity does not mean the reverse of varus deformity [**1**]. Total arthroplasty in a valgus knee is more difficult than those with varus deformities. Due to lateral capsuloligamentar retraction it is difficult to ensure a proper balance of the soft tissue in the frontal plane and adequate coverage of the bone surfaces In the presence of an important valgus deformity. The mostly demanding cases are those which display an ill-fated combination between an excessive lateral compartment bone wear, soft tissue retraction on the lateral side, soft tissue stretching on the medial side and, possibly, a history of a previous tibial osteotomy (**Fig. 1**).

This paper aims to present our experience in primary total knee arthroplasty for osteoarthritis of valgus knees, underlining the importance of a judicious balancing of the soft tissues around the joint.

## Materials and methods 

In the Department of Orthopedics and Traumatology, Bucharest University Hospital, between 2005 and 2007, 28 cases of primary total knee arthroplasty for osteoarthritis valgus knees were performed. .

 Preoperatively, the valgus angle of the knee was measured on a long leg anteroposterior (AP) X-ray view in standing position and the values were: 6 to15° in 14 cases, 15 to 25° in 10 cases and over 25° in 4 cases. The study group consisted of 21 women and 7 men with a mean age of 66.6 years (extremes 54-81).

 Capsuloligamentar balancing can be effective only if the bone cuts are done with respect to a few principles [**2**]. The first principle to be followed is that the bone cuts must be performed in such a way that the articular surface of the femoral prosthetic component is parallel to the femoral epicondyle line and the tibial component is perpendicular to the long axis of the tibia. Secondly, but equally important, is the principle of placing the femoral component in the appropriate external rotation, depending on the degree of the valgus deformity.

**Fig. 2:**
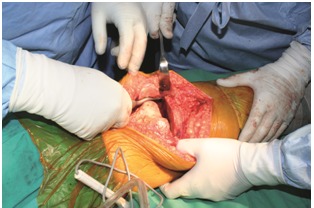
The release of the iliotibial tract and posterolateral corner from the tibia

 Soft tissue balancing of the knee in the frontal plane was achieved by: detaching the iliotibial tract from Gerdy’s tubercle, release of the lateral capsule from the posterolateral tibial corner, and detaching of the lateral collateral ligament and popliteal tendon from the lateral femoral epicondyle (**Fig. 2**). The mean postoperative follow-up period was of 14 months with a range between 7 and 29 months. Clinical and radiological evaluation were done regarding joint mobility, the KSS score and the femorotibial angle were measured on a long leg AP X-ray view in standing position.

## Results

 The average length of hospitalization was of 7-9 days. Only one patient was kept in hospital up to 18 days after surgery because of residual swelling in the lower third of the calf where he had a history of varicose ulcer. This complication was solved by elevating the limb and antibiotic treatment with cephalosporin and aminoglycoside.

 Joint mobility has improved from an average of 71° (extremes 52-87) preoperatively to an average of 95° (extremes 78-110) postoperatively. Two patients remained with an extension deficit of 10°. The first patient, who had a preoperative fixed valgus of 25° and an extension deficit of 15°, despite the correct postoperative alignment of the knee in frontal plane and posterior joint capsule release, continued to present this extension deficit at 12 months after surgery. The second one suffered of Alzheimer's disease and his cooperation with the physical therapy team was very difficult. The immobilization of the knee in an extension brace was even necessary, in between the rehabilitation sessions, in order to prevent the patient from keeping his knee in flexion. 

 The KSS mean score increased from 21.3 points (extremes 1-33) preoperatively to 80.7 points (extremes 70-92) postoperatively. All the 28 patients had a pain free knee when walking and resting. 

**Fig. 3:**
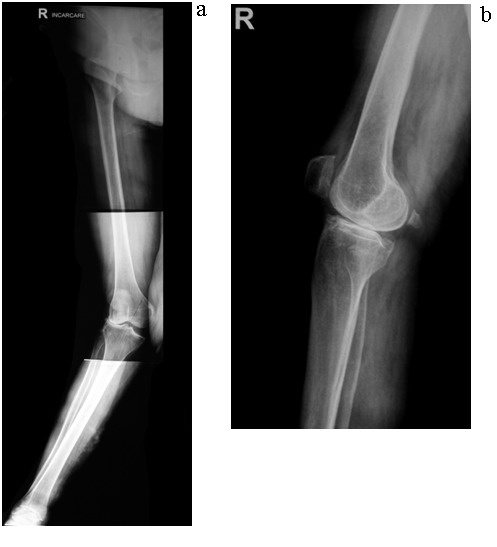
Preoperative long leg X-ray examination of the knee in standing position;AP view (a) and lateral view (b)

 Femorotibial angle was corrected from an average of 21° of valgus (extremes 11-39) preoperatively (**Fig. 3**) to a mean value of 9° of valgus (extremes 0-12) postoperatively. In 2 cases, due to bone deficiency in the lateral compartment, the tibial cuts were done in a valgus of 5 and respectively 7°. This technical trick did not alter the functional outcome.

 No other postoperative general or local complications were recorded in our study group.

## Discussions

 The approach for primary total knee arthroplasty on genu valgus may be medial or lateral. Advocates of the lateral approach claim that from the lateral side one can directly address to any soft tissue retractions [**3**], but once the release is done and the valgus corrected, there will be not enough soft tissue for joint closure and we face the risk of leaving part of the prosthesis under the skin. Approaching on the medial side, the tibial plateau must not be exposed and released more than its anteromedial half. The knee being lax in the medial compartment, we know from the beginning that we will not need any extended access to this compartment and we even risk increasing the preexisting laxity. Because the bone cuts should be performed perpendicular to the biomechanical axis of the femur and respectively tibia [**4**], in the medial compartment (convex aspect of the knee) a minimum resection must be done with a size not larger than the thickness of the prosthetic components that will be implanted. By contrary, in the lateral compartment (concave aspect of the knee), by an appropriate soft tissue release, we must create a rectangular space large enough to "fit" the prosthetic components. Overall, this soft tissue release on the lateral side is the release of the lateral collateral ligament and bone osteophytes. Often there is an external rotation of the tibial plateau accompanied by a shift in external rotation of the tibial tubercle and pronounced wear of the lateral tibial plateau in which the lateral femoral condyle seems to "sink". Lateral compartment bone surfaces undergo asymmetric wear in the sagittal plane, which explains why some soft tissues are tighter than others and why the joint laxity changes in different position of flexion. The level of the bone cuts must be appreciated with reference to the least damaged compartment [**5**]. Because the valgus deformity, the lateral femoral condyle can be hypoplastic or it can display a very advanced degree of wear; therefore the medial femoral condyle (less damaged) should be used as a landmark. Similarly, the lateral tibial plateau is often excavated and with significant changes in bone structure; that is why the probe guide that appreciates the level of the tibial bone cut must be applied on the medial tibial plateau.

 After the bone cuts are done correctly, capsuloligamentar balancing should be achieved, both in extension and in flexion position [**6**]. Lateral collateral ligament and popliteal tendon, due to their insertion close to the tibial rotation axis, will be strained both in flexion and extension. The iliotibial tract and the posterolateral capsule are relaxed in flexion but stretched in extension. Starting from these biomechanical findings we can state an algorithm of lateral release [**7**]. If the lateral compartment is tight only in extension but not in flexion, the iliotibial tract must be released first, from the Gerdy’s tubercle. If it still remains tight, posterolateral capsule is released completely. If the lateral compartment is tight in both flexion and extension, we must start by detaching the lateral collateral ligament and popliteal tendon from the lateral femoral condyle. In about half of the cases this actions are sufficient. If the knee remains tight in extension, the iliotibial tract must be released and if this is not enough posterolateral capsule should be detached, too. In case the lateral compartment is tight only in flexion but not in extension, the iliotibial tract must not be released because it relaxes in flexion anyway [**8**] but the lateral collateral ligament and posterolateral capsule should be detached. If the release is not enough it can be augmented by sectioning the intramuscular septum and lateral gastrocnemius insertion. Posterior cruciate ligament contributes substantially in maintaining the valgus deformity and, therefore, it must be frequently excised; this action makes necessary the implantation of a posterior stabilized prosthesis. 

 The higher is the preoperative valgus of the knee, after its correction by the prosthesis implantation the more important will be the extensor apparatus misalignment which will lead to patellar subluxation or patellar dislocation [**9**]. If the patient has an initial mild valgus (up to 9-10 degrees), the distal femoral cut is done at an angle of 7 degrees and this is enough to preserve the initial alignment of the extensor apparatus. If the preoperative valgus is higher, a lateral retinaculum release is required and in addition, a more medial placement of the patellar prosthetic component (in all these cases patellar resurfacing is mandatory). 

 In patients with significant valgus deformity there is a risk of paresis or paralysis of the common fibular nerve by elongation when the knee axis is restored to an anatomic position. As a precaution, in these patients spinal or epidural anesthesia should be avoided because it increases the risk of such complications and can hide them in the first hours following surgery. 

 Another consequence of the excessive preoperative valgus is medial collateral ligament elongation. In order to correctly balance the knee, a significant lateral release should be done, which will increase the distance between the femur and tibia ("extension gap") with the need of a thicker tibial insert implantation [**10**]. Due to this, the prosthetic knee space line will move more proximally in average with 5.5 mm without significant consequences on the femuropatellar joint. This is another reason for choosing a posterior stabilized prosthesis in these cases because it allows elevation of the joint space up to 10 mm, without functional consequences. 

 Before surgery, any deformities of the joints adjacent to the knee (hip and ankle) should be diagnosed [**11**]. Thus, dysplasia of the hip or congenital subluxation of the hip can lead to secondary genu valgus. Valgus flat foot is another reason for knee valgus deviation. Normally, these neighborhood deformities must be solved prior to knee arthroplasty in order to ensure a successful surgery with good functional results. 

## Conclusions

 Preoperative clinical and radiological evaluation of these cases is very important; long leg AP view X-ray examination in standing position is mandatory. 

 Standard medial parapatellar approach is appropriate in this type of arthroplasty even if significant knee valgus deviations are present because it avoids the lateral approach complications related to failure of soft tissue coverage at the time of wound closure. 

 Postoperatively one can get an aligned and stable knee if a judicious and progressive periarticular soft tissues balancing is achieved, in both flexion and extension position. 
